# Bruch’s-Mimetic Nanofibrous Membranes Functionalized with the Integrin-Binding Peptides as a Promising Approach for Human Retinal Pigment Epithelium Cell Transplantation

**DOI:** 10.3390/molecules27041429

**Published:** 2022-02-21

**Authors:** Shaocheng Wang, Siyong Lin, Bo Xue, Chenyu Wang, Nana Yan, Yueyan Guan, Yuntao Hu, Xuejun Wen

**Affiliations:** 1 Endocrine Department, Third Central Hospital of Tianjin, Tianjin 300170, China; shaocheng-wang@163.com (S.W.); seanlin1618@163.com (N.Y.); li12120502@163.com (Y.G.); 2 Tianjin Key Laboratory of Artificial Cell, Artificial Cell Engineering Technology Research Center of Public Health Ministry, Tianjin 300170, China; 3 Department of Chemical and Life Science Engineering, School of Engineering, Virginia Commonwealth University, Richmond, VA 23284, USA; siyonglin@163.com (S.L.); bxue@vcu.edu (B.X.); wangc6@vcu.edu (C.W.); 4 Department of Ophthalmology, Beijing Tsinghua Changgung Hospital, School of Clinical Medicine, Tsinghua University, Beijing 102218, China; 5 Key Laboratory of Spine and Spinal Cord Injury Repair and Regeneration of Ministry of Education, Orthopaedic Department of Tongji Hospital, School of Life Science and Technology, Tongji University, Shanghai 200065, China; 6 International Institute for Biomedical Biomaterials (I^2^BM), Zhengzhou 450018, China

**Keywords:** retinal pigment epithelial transplantation, integrin, peptides, electrospinning, Bruch’s membrane, immunogenicity, cell adhesion, nanofibers

## Abstract

Background: This study aimed to develop an ultrathin nanofibrous membrane able to, firstly, mimic the natural fibrous architecture of human Bruch’s membrane (BM) and, secondly, promote survival of retinal pigment epithelial (RPE) cells after surface functionalization of fibrous membranes. Methods: Integrin-binding peptides (IBPs) that specifically interact with appropriate adhesion receptors on RPEs were immobilized on Bruch’s-mimetic membranes to promote coverage of RPEs. Surface morphologies, Fourier-transform infrared spectroscopy spectra, contact angle analysis, Alamar Blue assay, live/dead assay, immunofluorescence staining, and scanning electron microscopy were used to evaluate the outcome. Results: Results showed that coated membranes maintained the original morphology of nanofibers. After coating with IBPs, the water contact angle of the membrane surfaces varied from 92.38 ± 0.67 degrees to 20.16 ± 0.81 degrees. RPE cells seeded on IBP-coated membranes showed the highest viability at all time points (Day 1, *p* < 0.05; Day 3, *p* < 0.01; Days 7 and 14, *p* < 0.001). The proliferation rate of RPE cells on uncoated poly(ε-caprolactone) (PCL) membranes was significantly lower than that of IBP-coated membranes (*p* < 0.001). SEM images showed a well-organized hexa/polygonal monolayer of RPE cells on IBP-coated membranes. RPE cells proliferated rapidly, contacted, and became confluent. RPE cells formed a tight adhesion with nanofibers under high-magnification SEM. Our findings confirmed that the IBP-coated PCL membrane improved the attachment, proliferation, and viability of RPE cells. In addition, in this study, we used serum-free culture for RPE cells and short IBPs without immunogenicity to prevent graft rejection and immunogenicity during transplantation. Conclusions: These results indicated that the biomimic BM-IBP-RPE nanofibrous graft might be a new, practicable approach to increase the success rate of RPE cell transplantation.

## 1. Introduction

Degenerative retinal diseases such as age-related macular degeneration (AMD) or retinitis pigmentosa (RP) are the leading cause of irreversible vision loss in people over 50 [1,2,3,4]. Although these diseases affect so many people around the world, no basic measures are available to mitigate their progression. Retinal pigment epithelium (RPE) cell dysfunction or loss is one of the main pathological changes leading to such a wide range of degenerative retinal diseases. RPE cells are essential for maintaining normal physiology in the neurosensory retina and photoreceptors. Loss or damage of RPE cells can cause photoreceptor dysfunction and irreversible blindness [5,6,7].

RPE cell transplantation has been proposed as one of the most promising strategies to replenish or replace damaged or lost RPE cells. However, few patients have achieved significant visual improvement through RPE cell transplantation [8,9]. The inability of transplanted RPE cells to adhere and proliferate on Bruch’s membrane (BM) is the main reason for the failure of RPE cell transplantation [10,11,12].

Furthermore, in degenerative retinal diseases, the Bruch’s membrane (BM) undergoes various pathological changes due to chronic inflammation, abnormal production of extracellular matrices, and abnormal neovascularization, which result in the inability of BM to support RPE cell adhesion. On the other hand, damaged BMs do not act as permeable molecular sieves to regulate the balanced exchange of nutrients, biomolecules, oxygen, and metabolic waste between the retina and choroid [13,14]. Given that BMs play a vital role in maintaining the physiological functions of RPE cells, it is therefore critical to create biomimic scaffolds that can replace diseased BMs while supporting RPE cells to adhere and survive.

Among various fabrication technologies, electrospinning has emerged as an exciting means to generate nanofibrous membranes mimicking natural extracellular matrices (ECMs), as well as the porous structure of BMs, and enhance cell infiltration and effective exchange of nutrients and metabolites [15,16]. Poly(ε-caprolactone) (PCL) has become a widely used synthetic polymer because of its excellent biodegradability, biocompatibility, and mechanical properties. Several medical devices made of PCL have been approved by the US Food and Drug Administration (FDA) for clinical applications [17,18]. Electrospun nanofibrous structures have also been used to develop various biomedical applications such as drug delivery, implants, prosthetic devices, and tissue engineering scaffolds in our group [19,20,21,22]. Although PCL-based electrospun nanofibrous membranes exhibit desirable degradable and mechanical characteristics, the inherent hydrophobic nature of PCL limits tissue engineering applications due to poor cell adhesion [23,24]. Enhancing adherence and survival of RPE cells to the biomimic BM has become a successful strategy for retinal tissue regeneration.

Mousa et al. found that the attachment of RPE cells to BM is mainly mediated by the interaction between the integrins on the surface of RPE cells and the ligands in the ECM [25]. Integrins represent the most important family of cell–ECM adhesion receptors. Integrins are not only adhesion “hooks”, but they also send critical signals to cells about the nature of their surroundings when cells decide which biological actions to take (e.g., adhesion, proliferation, migration, or death) [26,27,28,29]. Therefore, integrins are at the core of many cell biological processes. The importance of integrins in RPE cell transplantation has been demonstrated by several researchers [30,31,32]. Zarbin showed that the lack of expression of integrins necessary for attachment in freshly harvested RPE cells may account for the poor adhesion of BM [33]. Engineering biomaterials with specific integrin-binding activity might be an effective and practicable approach that can promote cell adhesion and induce biological responses.

Several short peptides have been shown to support specific integrin-binding with a variety of cells [34,35,36]. Previously, our laboratory developed a peptide-based coating matrix for human neural stem cells [37]. In this study, the short and bioactive integrin-binding peptides (IBPs) used were specific a_5_b_1_ integrin-binding peptides with 12 amino acids. To further facilitate RPE cell adhesion and proliferation, we modified the electrospun nanofibrous membrane with short, specific a_5_b_1_ integrin-binding peptides using thiol-vinyl sulfone click chemistry (PVA-VS). We then investigated the physical, chemical, and cytocompatibility properties of biomimic BM-IBP-RPE nanofibrous membranes.

## 2. Results

### 2.1. Characterization of Electrospun Nanofibrous Membranes

**Figure 1:**
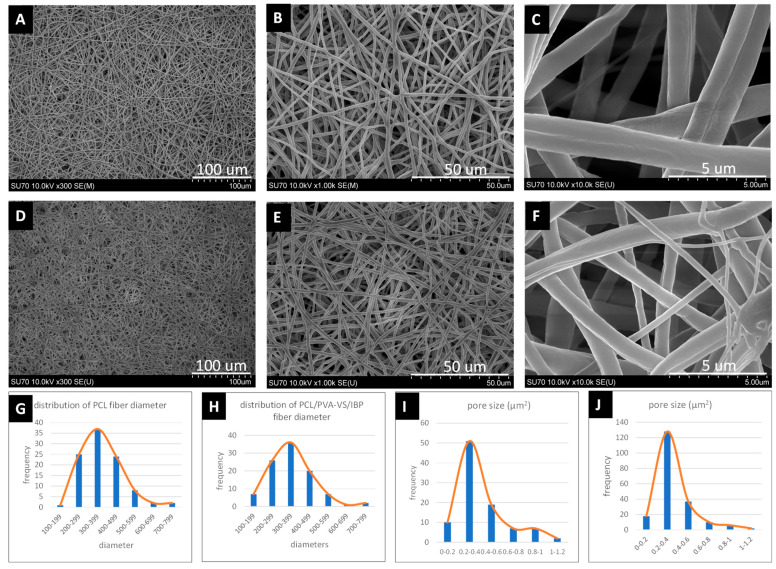
Surface characterization of PCL and IBP-coated nanofibrous membranes. (**A**) PCL nanofibrous membranes, ×300; (**B**) PCL nanofibrous membranes, ×1.00k; (**C**) PCL nanofibrous membranes, ×10k; (**D**) IBP-coated nanofibrous membranes, ×300; (**E**) IBP-coated nanofibrous membranes, ×1.00k; (**F**) IBP-coated nanofibrous membranes, ×10k. SEM images show morphologies of samples with random fibrous orientation and variably sized pores. Average diameter of IBP-coated membranes is not significantly different from uncoated PCL membranes, and pore sizes of IBP-coated membranes were also similar to uncoated PCL membranes. Distribution of fiber diameters for PCL (**G**) and IBP-coated (**H**) nanofibrous membranes. Pore sizes for PCL (**I**) and IBP-coated (**J**) nanofibrous membranes.

SEM images of the nanofibrous membranes are shown in Figure 1, including images of electrospun PCL membranes without coating (Figure 1A–C) and IBP-coated membranes (Figure 1D–F). Both types of membranes were composed of randomly oriented fibers and pores of different sizes and structures. The average diameter of uncoated PCL and IBP-coated PCL nanofibrous membranes was 376.18 ± 79.35 nm and 359.04 ± 86.15 nm, respectively. No significant difference in fiber diameter between uncoated and IBP-coated PCL membranes was found (*p* > 0.05) (Figure 1G,H). High-magnification images showed that the surface morphologies of the two nanofibrous membranes were very similar (Figure 1C,F). In addition, the average pore size (0.36 ± 0.17 μm^2^) of IBP-coated membranes was also similar to that of uncoated PCL membranes (0.39 ± 0.21 μm^2^, *p* > 0.05) (Figure 1I,J) (Table 1). When PCL was dissolved in HFIP at a concentration of 8% (wt/vol), the distance from the needle tip to the working collector was 15 cm, the high potential was 10 kV, the solution was fed at a rate of 25 μL/min, and the electrospinning time was 25 min to produce nanofibrous membranes with a thickness of 2.16 ± 0.78 μm, which is similar to that of native BM (1–4 μm).

### 2.2. Contact Angle Analysis

**Figure 2:**
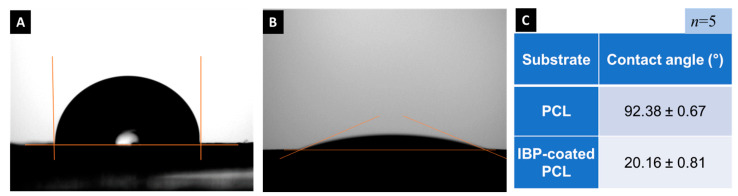
Contact angle analysis was performed to determine wettability of different membranes, including (**A**) PCL and (**B**) IBP-coated membranes. (**C**) Result indicated that hydrophilicity of PCL membranes was improved with IBP coating.

Contact angle analysis was performed to determine the wettability of the two groups of nanofibrous membranes (Figure 2). A lower contact angle value indicates that the water has a tendency to spread and adhere to the surface of the membrane, while a higher contact angle value indicates that the membrane has a tendency to repel water. After IBP coating, the water contact angle of the membranes varied from 92.38 ± 0.67 degrees (hydrophobic) to 20.16 ± 0.81 degrees (hydrophilic) (Figure 2A,B). This analysis indicated that the hydrophilicity of IBP-coated PCL membranes was much higher than that of PCL (*p* < 0.01) (Figure 2C), indicating that the coating of integrin-binding peptides significantly improved the hydrophilicity of nanofibrous membranes.

### 2.3. Chemical Composition of Nanofibrous Membranes

**Figure 3:**
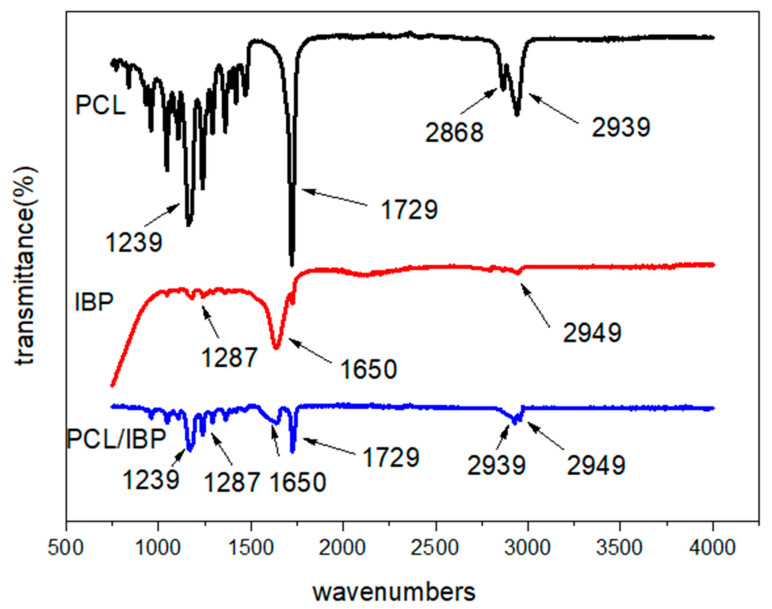
Fourier-transform infrared spectra (FTIR) of PCL membranes, IBP powder, and IBP-coated PCL membranes.

Fourier-transform infrared spectroscopy (FTIR) was used to identify different types of chemical bonds present in the samples. Figure 3 shows the characteristic bands of PCL, such as 2939 cm^−1^ (asymmetric CH_2_ stretching), 2868 cm^−1^ (symmetric CH_2_ stretching), 1729 cm^−1^ (ester carbonyl stretching), and 1239 cm^−1^ (asymmetric COC stretching) from both uncoated PCL and IBP-coated PCL nanofibrous membranes. Furthermore, IBP-coated nanofibrous membranes showed additional bands for amide I (1650 cm^−1^) and amide III (1287 cm^−1^) (Figure 3), which are typical peaks of a_5_b_1_ integrin-binding peptides. This result indicated that the a_5_b_1_ integrin-binding peptides were successfully immobilized on PCL nanofibrous membranes.

### 2.4. Biocompatibility Evaluation of Nanofibrous Membranes

**Figure 4:**
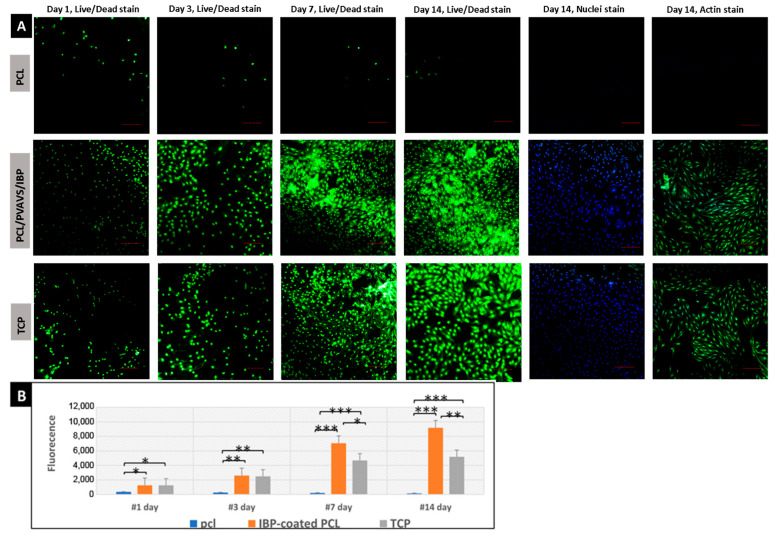
Biocompatibility of PCL, IBP-coated membranes, and TCP to ARPE-19 cells. (**A**) ARPE-19 cell morphology after growing on different groups for 1, 3, 7, and 14 days observed by laser scanning confocal microscopy. ARPE-19 cells were treated with live/dead viability test, and on Day 14, cells actin filament and nuclei were stained with AlexaFluor 488/546 Phalloidin and DAPI, respectively. Scale bars represent 200 μm. (**B**) ARPE-19 cells attached to different groups at comparable densities on Days 1, 3, 7, and 14 by Alamar Blue assay. ARPE-19 showed highest cell activity on IBP-coated membranes. Significant difference is denoted by: *** = *p* < 0.001, ** = 0.001 ≤ *p* ≤ 0.01, * = 0.01 ≤ *p* ≤ 0.05.

The RPE cell responses to nanofibrous membranes were used to evaluate the cytocompatibility of IBP coating. RPE cells viability and proliferation on PCL membrane samples, IBP-coated PCL membrane samples, and TCP were examined at 1, 3, 7, and 14 days. Figure 4A shows the cell density increased with culture time on IBP-coated membranes, as well as on TCP. However, the proliferation rate of RPE cells on uncoated PCL nanofibrous membrane was very low, and as time passed, the number of cells decreased. The RPE cells proliferated rapidly from Days 1 to 7 on both IBP-coated PCL membranes and TCP and exhibited better cell viability than uncoated PCL membranes (Day 1, *p* < 0.05; Day 3, *p* < 0.01; Day 7, *p* < 0.001).

In addition, IBP-coated PCL membranes yielded the highest cell density at all time points. Even when the TCP group reached confluence, the number of cells on IBP-coated fibrous membranes was still significantly higher than that of the TCP and uncoated PCL groups on Day 14 (*p* < 0.001). On Day 14, most surfaces of IBP-coated PCL membranes were covered with dense RPE cells, and some pores in the nanofibrous membrane were also filled with cells. The metabolic activity of RPE cells grown on TCP was similar to that of the IBP-coated PCL membrane in the early time points (*p* > 0.05), but it began to decline at around 14 days, which is consistent with the Alamar Blue data in Figure 4B.

### 2.5. SEM for Cell Morphology

**Figure 5:**
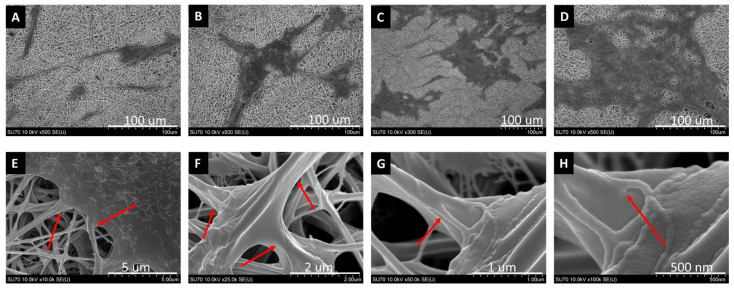
SEM images of RPE cells on the IBP-coated PCL nanofibrous membranes. (**A**–**D**) RPE cells growth on membranes on Days 1, 3, 7, and 14. The RPE cells formed a striking in vivo-like regional monolayer on Day 14 (**D**). With the help of integrin-binding peptides, the RPE cells formed a very tight connection with nanofibers (**E**–**H**), and the red arrows indicated the tight junctions.

As shown in Figure 5, under SEM observation, RPE cells attached quickly onto the IBP-coated PCL nanofibrous membranes. Encouragingly, RPE cells adhered to IBP-coated membranes, proliferated rapidly, and formed densely packed cell colonies within a week (Figure 5A–C). RPE cells grew well on IBP-coated membranes, and large areas of local confluence cell islands were seen. RPE monolayer formed tight junctions with nanofibers around Day 14 (Figure 5D). In contrast, very few RPE cells attached to uncoated PCL membranes. As the culture time increased, it was more difficult to find RPE cells on the uncoated PCL nanofiber membrane. Overall, RPE cells expand rapidly on the IBP-coated nanofibrous membranes, and the formation of connections between RPE cells and nanofibers can be clearly seen under high-magnification SEM (Figure 5E–H).

## 3. Discussion

RPE cell transplantation holds great promise in the treatment of AMD or RP. However, clinical experience with RPE transplants has been disappointing so far due to their low viability and high propensity to form aggregates rather than spreading monolayer cell sheets [38,39,40]. Our design was to use nonimmunogenic, degradable nanofibrous membranes on which RPE cells were pre-seeded after surface modification to support maturation into monolayers mimicking RPE/Bruch’s-mimetic membrane complexes. Such RPE/Bruch’s-mimetic membrane complexes enable easy and precise delivery.

Tezel et al. found that most RPE cells died shortly after transplantation, and RPE cells did not migrate out from the implantation site. The lack of adhesion of RPE cells on BM after transplantation might be the main cause of cell death because the cells cannot obtain the necessary adhesive signals to avoid cell death and initiate migration and proliferation [41,42,43]. Accumulating evidence points to the importance of integrins in the growth and survival of RPE transplants [30,31,32]. Gullapalli and Zarbin have shown that RPE cells express multiple integrin subunits, which allow RPE cells to interact with laminin, vitronectin, and collagen type II within the basal lamina [32,33]. Considering the key role of integrins, the lack of attachment of RPE cells after transplantation can be overcome by upregulating the expression of integrins and providing integrin-binding sites.

Since cell adhesion depends to a large extent on the interactions between cells and ECMs, we applied short peptides with high cell-binding activity, such as integrin-binding peptides, to mimic the binding between integrins and ECM. Short peptides have been used to functionalize various synthetic biomaterials with the purpose of instructing cell adhesion processes to improve the biological activity of synthetic biomaterials. In this study, we used novel short a_5_b_1_ integrin-binding peptides with 12 amino acids synthesized in our laboratory to support the adhesion and proliferation of RPE cells. The RPE cell-binding activity of the a_5_b_1_ integrin-binding peptide is similar to the full length of laminin and type II collagen, but compared with the intact protein, it is more stable, easier to synthesize, and less likely to exhibit steric hindrance. Surprisingly, few reports are available on the use of highly bioactive integrin-binding peptides as surface coating molecules to support RPE cell transplantation. This may be attributed to the fact that its development requires expertise in synthetic chemistry, but in fact, many of its excellent properties are particularly suitable for supporting biomaterials that promote human cell transplantation.

The immobilization of biologically active peptides (such as IBPs) on the surfaces of synthetic biomaterial surfaces represents key progress in surface biofunctionalization. We developed a readily applicable coating system to secure IBP molecules on the surface of electrospun PCL nanofibrils with the help of thiol-based click chemistry. PVA-VS is used to functionalize PCL nanofibrils with active vinyl sulfone groups, and integrin-binding peptides with thiol groups can be tightly connected to PVA-VS with the help of PEGTA. The thiol-VS bond has no effect on the biological activity of IBP molecules [44,45,46]. The result proved that the IBP-PVA-VS coating method is simple and efficient. Not only did it not damage the original nanofibril structure of the degradable PCL membranes, but also efficiently improved the hydrophilicity and bioactivity of the PCL membrane.

Firstly, it should be pointed out that the use of thiol-based click chemistry to immobilize IBPs on PCL nanofibrous membranes will not change the original morphologies of the PCL nanofiber microstructure. The average diameters of uncoated PCL and IBP-coated PCL nanofiber membranes are not significantly different from each other, and high-magnification images showed that their surface morphologies are similar. The selective reciprocal exchange and transfer of nutrients, biomolecules, oxygen, and metabolic waste are the key functions provided by healthy native BMs [47,48], and these functions can also be provided by our Bruch’s-mimetic membranes because of their proper pore sizes. We also controlled the electrospinning parameters to ensure that the thickness of the nanofibrous membrane (2.16 ± 0.78 μm) is similar to that of native BMs [48,49]. Moreover, compared with the limitations of the two-dimensional flat surface of TCP, IBP-coated nanofiber membranes can improve cell adhesion and proliferation by providing more cell attachment sites in three-dimensional organization. Therefore, the IBP-PVA-VS-PCL nanofiber membranes we created are excellent scaffolds to replace diseased BMs.

Secondly, in this study, by identifying specific absorption bands, FTIR spectra confirmed that IBPs were successfully coated or immobilized on the PCL nanofibrous membranes. At the same time, the nanofibril membranes coated with IBPs did not show any changes in the characteristic peaks of PCL and IBP peptides, which may explain why the biological activities of integrin-binding peptides were not affected after coating.

Thirdly, according to the results of this study, after IBP coating, the nanofibrous membranes showed rapid penetration of water drops into the membranes. The water contact angle of the membranes changed from 92.38 ± 0.67 degrees (hydrophobicity) for uncoated PCL membranes to 20.16 ± 0.81 degrees (hydrophilic) for IBP-coated PCL membranes. The hydrophilicity of nanofiber membranes is an important physical parameter because it can directly affect the probability of cell adhesion. Our findings indicated that the IBP-PVA-VS coating method we developed successfully changed the hydrophilicity of the membranes.

So how to develop such IBP-PVA-VS-PCL nanofiber grafts loaded with live RPE? After the manufacture of BM substitutes and surface biofunctionalization, it is necessary to evaluate whether RPE cells can adhere to Bruch’s-mimetic membranes, as well as the proliferation of RPE cells on the membranes. In our study, RPE cells on IBP-coated membranes showed the highest viability and cell number at all time points compared with the uncoated PCL membrane control and the TCP control. The proliferation rate of RPE cells on uncoated PCL nanofibrous membranes was significantly lower than that of IBP-coated membranes and TCP flat surfaces. The role of IBP coating not only changed the surfaces of PCL from hydrophobic to hydrophilic, but more importantly, it also promoted the adhesion and spreading of RPE cells in a serum-free environment [50,51,52]. Thus, the function of IBPs present in Bruch’s-mimetic membranes was critical in developing stable surfaces for RPE integration with nanofibrous membranes.

The SEM image of RPE cells grown on the IBP-coated membranes also showed that the well-organized hexa/polygonal monolayer RPE cells proliferated rapidly, contacted each other, and became confluent. High-power SEM images showed that RPE cells formed connections with nanofibers. This result proved that the PCL membrane coated with IBPs maintains the stable adherence and health of RPE cells.

For clinical cell transplantation, ensuring the low immunogenicity of the carrier biomaterial is more important to the success of transplants. Our research prepared for this from two aspects: On the one hand, in most studies, animal- or human-derived biomolecules (such as Matrigel or Laminin) have been widely used as coating molecules. However, these molecules introduce immunogenicity to the graft and raise safety concerns for human clinical use due to immunogenicity and potential pathogens transfer [53,54,55,56]. This is why we have been trying to develop coatings without immunogenicity by using pure synthetic small molecules. Short peptides (less than 15 amino acids) are promising candidates. Using peptide synthesizers, peptides of this size can be easily synthesized using animal-free ingredients in our laboratory. In addition, short peptides of this size also have great advantages, including good biocompatibility, nontoxicity, and inherent biodegradability, so they are able to greatly improve the safety of clinical transplantation [34,35,36,37]. On the other hand, xeno-free and serum-free culture is the essential condition for preparing RPE transplants for human clinical use. Although RPE cells can adhere and survive on PCL membranes in serum-containing cultures, there is little research on the adhesion and proliferation of RPE cells on the PCL membrane in serum-free and xeno-free cultures. Our results confirmed that it is difficult for RPE cells to survive, adhere, and spread on the PCL membrane under serum-free and xeno-free culture conditions; however, the surface modification of membranes with integrin-binding peptides helped RPE cells successfully address this problem.

In short, biomimetic BM-IBP-RPE membranes exhibited the following characteristics: larger surface area with appropriate porosity and pore size, a suitable thickness of 2.16 ± 0.78 μm, cell adhesion sites, and nonimmunogenicity under xeno-free and serum-free culture conditions. Thus, this strategy can be used to develop a new generation of grafts for transplant applications in tissue engineering and regenerative medicine.

## 4. Materials and Methods

### 4.1. Materials and Supplies

Poly(Ɛ-caprolactone) (PCL, *M*_n_ = 80,000), glutaraldehyde, dimethyl sulfoxide (DMSO), phosphate-buffered saline (PBS), Hank’s balanced salt solution (HBSS), antibiotics (penicillin–streptomycin–fungizone), and paraformaldehyde were ordered from Sigma-Aldrich (St. Louis, MO, USA). Hexafluoro-2-propanol (HFIP) was acquired from Oakwood Inc. (West Columbia, SC, USA). Thiolated a_5_b_1_ integrin-binding peptides, CellPhilic™ xeno-free and serum-free culture medium for human epithelial cells (SKU: ESC-10×50 mL), vinylsulfone-functionalized polyvinyl alcohol (PVA-VS), and 4-arm polyethylene glycol acrylate (PEGTA) were ordered from Biomaterials USA LLC (Richmond, VA, USA). LIVE/DEAD™ Reduced Biohazard Cell Viability Kit, AlexaFluor 488/546 Phalloidin, and 4′-6-diamidino-2-phenylindole (DAPI) were purchased from ThermoFisher (Carlsbad, CA, USA). Alamar Blue™ reagent was obtained from Bio-Rad Laboratories (Hercules, CA, USA). The adult human retinal pigmented epithelium-19 (ARPE-19, ATCC-CRL-2302™) cell line was purchased from American Type Culture Collection (Manassas, VA, USA).

### 4.2. Electrospinning of Porous Ultrathin Nanofibrous Membranes

Poly(Ɛ-caprolactone) particles were dissolved in HFIP at 8% (wt/vol) concentration and stirred overnight to obtain a homogenous solution. A 5 mL syringe was connected to a 23 G blunt-ended needle that served as the charged spinneret. A high-voltage power supply (Gamma High Voltage Research, Ormond Beach, FL, USA) was connected between the needle tip and the custom-made collector and served as a ground to generate a high electrical potential of 10 kV, and nanofibrous membranes were formed between the two metal plates. The distance between the needle tip and the collector was 15 cm, and the polymer solution was fed at a rate of 25 μL/min to obtain bead-free fibrous membranes. All electrospinning experiments were performed at a constant temperature of 25 ± 1 °C and relative humidity of 20 ± 5%. The thickness of the nanofibrous membrane was also an important aspect in the design of BM substitutes. In order to find electrospinning conditions able to achieve the thickness of the nanofiber membrane equivalent to that of native BM, we conducted electrospinning experiments by changing the electrospinning time while keeping all other parameters constant. The electrospun membranes were placed under vacuum for 7 days to eliminate organic solvent residues and then carefully secured on thin 316 L medical-grade stainless-steel rings of 25 mm inner diameter for further studies.

### 4.3. Modification of Nanofiber Membranes with IBPs

**Scheme 1:**
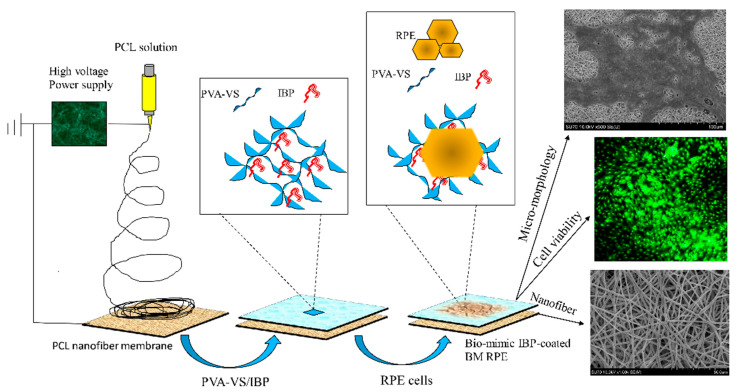
Fabrication of biomimic, IBP-coated, BM-mimicking nanofibrous membranes.

PCL nanofibrous membranes were sterilized under ultraviolet light for 1 h on both sides. After soaking with sterile distilled water overnight and 0.01 mg/mL solution of vinylsulfone-functionalized polyvinyl alcohol (PVA-VS) for 15 min at room temperature, the membranes were washed with sterile PBS 3 times, then immersed in sterile 0.125 mg/mL thiolated a_5_b_1_ integrin-binding peptide solution for 2 h to immobilize the integrin-binding peptides on the surfaces of nanofibrous membranes through thiol-vinyl sulfone click chemistry. All these procedures were performed under sterile environment. Coated nanofibrous membranes were directly used for cell culture experiments or dried before future use. The manufacturing process is illustrated in Scheme 1.

### 4.4. Surface Morphology Characterization

The morphology and diameters of naked PCL and IBP-coated PCL nanofibrous membranes were examined with a field-emission scanning electron microscopy system (FE-SEM, Hitachi SU-70, Tokyo, Japan). Twelve samples were coated with a mixture of palladium and gold (Pd/Au = 40:60) before SEM observation. Micrographs of each sample were obtained at random locations. The diameter and pore size of electrospun nanofibers were measured using ImageJ analysis software (ImageJ 1.42q, National Institute of Health, Bethesda, MD, USA). The mean and standard deviation of the fiber diameter and pore size data were collected from at least 120 fibers from each sample.

### 4.5. Surface Wettability Measurement

The most common method for characterizing surface wettability is sessile drop goniometry. This method determines the contact angle according to the shape of the droplet and can be applied to a wide variety of materials. The wettability of PCL and IBP-coated nanofibrous membranes was evaluated according to the water surface contact angle by the sessile drop method using 1 μL of deionized water that was dropped onto the surfaces of nanofibrous membranes. After 10 s, the shapes of the water droplets were recorded by an optical contact angle meter system (OCA20, Data Physics Instruments GmbH, Filderstadt, Germany). The measurements were performed on five different areas of the surfaces, and the values were averaged.

### 4.6. Fourier-Transform Infrared Spectroscopy (FTIR) Analysis

The chemical structures of nanofibrous membranes were analyzed by an FTIR spectroscopy system (NICOLET iS10, Thermo Scientific, Waltham, MA, USA). The infrared spectra of samples were measured over a wavelength range of 400–4000 cm^−1^.

### 4.7. Cell Culture

Adult human retinal pigmented epithelium-19 (ARPE-19) cells were cultured in CellPhilic™ xeno-free and serum-free culture medium for human epithelial cells. Cells were maintained at 37 °C in a humidified incubator controlled at 5% CO_2_ atmosphere. The medium was changed twice a week, and ARPE-19 cells between Passages 6 and 10 were used for our experiments.

### 4.8. Biocompatibility Test

#### 4.8.1. ARPE-19 Proliferation Assay

ARPE-19 cells at Passage 6 were utilized to test the in vitro biocompatibility of PCL and IBP-coated nanofibrous membranes. Tissue culture polystyrene (TCP) served as control. Sterilized nanofibrous membranes attached to 316 L stainless-steel rings of 32 mm diameter were placed in 6-well cell culture plates and conditioned with xeno-free and serum-free culture medium for 12 h before cell seeding. Then, 1 × 10^5^ cells per sample were seeded on each nanofibrous membrane. To investigate cell proliferation, ARPE-seeded membranes were incubated for 1, 3, 7, and 14 days. The culture medium was replaced every 2 days. Cell growth was observed using an optical fluorescent microscope (CKX 41, Olympus, Shinjuku-ku, Japan).

#### 4.8.2. Alamar Blue Assay for Cell Viability

Alamar Blue assay was used to determine the viability of ARPE-19 cells on the PCL and IBP-coated PCL nanofibrous membranes and TCP. On Days 1, 3, 7, and 14, 10% Alamar Blue in culture medium was used to determine cell viability. After culturing with 10% Alamar Blue in the dark for 4 h before fluorescence intensity reading with a microplate reader (Synergy H1 Hybrid reader, BioTek, Miami, FL, USA), the excitation wavelength and emission wavelength were set at 540 nm and 570 nm, respectively. All experiments were run in triplicate.

#### 4.8.3. Live/Dead Staining

To observe the cell viability of each group, ARPE-19 cells were processed with the LIVE/DEAD Reduced Biohazard Cell Viability Kit following the manufacturer’s instructions on Days 1, 3, 7, and 14. Briefly, each group was washed with equivalent HBSS twice before processing with live/dead reagents for 15 min at room temperature. Then, 4% freshly prepared glutaraldehyde in HBSS was used to fix ARPE-19 cells for 1 h. The fluorescence images were observed under a confocal laser scanning microscope (IX81, Olympus, Shinjuku-ku, Japan).

#### 4.8.4. Immunofluorescence Staining

After 14 days of culture on the PCL and IBP-coated PCL nanofibrous membranes and TCP, RPE cells were fixed and stained with AlexaFluor 488/546 Phalloidin for the actin filament inside the cells and DAPI for the cell nuclei according to the manufacturer’s protocol. Briefly, 4% paraformaldehyde was used to fix the RPE cells on samples for 30 min, and the samples were washed with PBS solution three times. The actin filaments in cells were dyed for 40 min, and the nuclei were treated with DAPI for 20 min. Finally, the stained samples were transferred into a confocal dish that was mounted before being evaluated. Confocal laser scanning microscopy (Olympus IX81, Japan) was used to image the cytoskeletal and nuclear organizations of RPE cells cultured under different experimental conditions.

#### 4.8.5. Scanning Electron Microscopic Analysis

The micromorphology of ARPE-19 cells seeded on electrospun Bruch’s-mimetic nanofibrous membranes was evaluated using scanning electron microscopy (SEM). The samples were prepared for SEM examination on Days 1, 3, 7, and 14. In brief, the cell-seeded samples were gently washed with warm PBS and fixed with 2.5% glutaraldehyde in PBS solution for 24 h at 4 °C. Then, they were dehydrated in graded ethanol solutions (30%, 50%, 70%, 90%, and 100%) and vacuum-dried overnight. All samples were sputter coated with palladium and gold, and images were taken with a scanning electron microscopy (SEM) system (SU-70, Hitachi, Tokyo, Japan) at an accelerating voltage of 15 kV.

### 4.9. Statistical Analysis

Statistical analyses were performed using one-way analysis of variance (ANOVA) by PASW Statistics Version 18 (SPSS Inc., San Francisco, CA, USA), followed by Tukey’s post hoc tests and the paired *t*-test, as appropriate. Results were presented as mean ± SD. A value of *p* < 0.05 was considered statistically significant.

## 5. Conclusions

In summary, in this study, we developed ultrathin nanofibrous membranes that mimic the native architecture of BM. Furthermore, coating with integrin-binding peptides improved the attachment, growth, and proliferation of RPE cells. This novel biomimic BM-IBP-RPE nanofibrous graft might be a new practicable method to increase the success rate of RPE cell transplantation in the treatment of AMD or RP.

## Figures and Tables

**Table 1:** Physical properties of PCL and IBP-coated PCL membranes (n = 120).

Nanofibrous Membranes	Fiber Diameter (nm)	Pore Size (μm^2^)
PCL	376.18 ± 79.35	0.39 ± 0.21
IBP-coated PCL	359.04 ± 86.15	0.36 ± 0.17

## Data Availability

Not applicable.

## Sample Availability

Samples of the compounds are available from the authors.

## Abbreviations

RPE	Retinal pigment epithelial
BM	Bruch’s membrane
IBP	Integrin-binding peptides
AMD	Age-related macular degeneration
RP	Retinitis pigmentosa
ECM	Extracellular matrix
PCL	Poly(ε-caprolactone)
PVA-VS	Vinylsulfone functionalized polyvinyl alcohol
PEGTA	4-Arm polyethylene glycol acrylate
HFIP	Hexafluoro-2-propanol
FE-SEM	Field-emission scanning electron microscopy
TCP	Tissue culture polystyrene
FTIR	Fourier-transform infrared spectroscopy

## References

[1] Thomas C.J., Mirza R.G., Gill M.K. (2021). Age-Related Macular Degeneration. Med. Clin. N. Am..

[2] Bowes Rickman C., LaVail M.M., Anderson E.R., Grimm C., Hollyfield J., Ash J. (2016). Retinal Degenerative Diseases: Mechanisms and Experimental Therapy.

[3] Wong C.W., Yanagi Y., Lee W.K., Ogura Y., Yeo I., Wong T.Y., Cheung C.M.G. (2016). Age-related macular degeneration and polypoidal choroidal vasculopathy in Asians. Prog. Retin. Eye Res..

[4] Day S., Acquah K., Lee P.P., Mruthyunjaya P., Sloan F.A. (2011). Medicare costs for neovascular age-related macular degeneration, 1994–2007. Am. J. Ophthalmol..

[5] Zarbin M.A. (2004). Current concepts in the pathogenesis of age-related macular degeneration. Arch. Ophthalmol..

[6] Ding X., Patel M., Chan C.C. (2009). Molecular pathology of age-related macular degeneration. Prog. Retin. Eye Res..

[7] Campbell M., Humphries P. (2012). The blood-retina barrier: Tight junctions and barrier modulation. Adv. Exp. Med. Biol..

[8] Qiu T.G. (2019). Transplantation of human embryonic stem cell-derived retinal pigment epithelial cells (MA09-hRPE) in macular degeneration. NPJ Regen. Med..

[9] Binder S., Stolba U., Krebs I., Kellner L., Jahn C., Feichtinger H., Povelka M., Frohner U., Kruger A., Hilgers R.-D. (2002). Transplantation of autologous retinal pigment epithelium in eyes with foveal neovascularization resulting from age-related macular degeneration: A pilot study. Am. J. Ophthalmol..

[10] Pilotto E., Midena E., Longhin E., Parrozzani R., Frisina R., Frizziero L. (2019). Müller cells and choriocapillaris in the pathogenesis of geographic atrophy secondary to age-related macular degeneration. Graefes Arch. Clin. Exp. Ophthalmol..

[11] McHugh K.J., Tao S.L., Saint-Geniez M. (2014). Porous poly(ε-caprolactone) scaffolds for retinal pigment epithelium transplantation. Investig. Ophthalmol. Vis. Sci..

[12] Shirai H., Mandai M., Matsushita K., Kuwahara A., Yonemura S., Nakano T., Assawachananont J., Kimura T., Saito K., Terasaki H. (2016). Transplantation of human embryonic stem cell-derived retinal tissue in two primate models of retinal degeneration. Proc. Natl. Acad. Sci. USA.

[13] Curcio C.A., Johnson M. (2013). Structure, function, and pathology of Bruch’s membrane. Elastic.

[14] Booij J.C., Baas D.C., Beisekeeva J., Gorgels T.G., Bergen A.A. (2010). The dynamic nature of Bruch’s membrane. Prog. Retin. Eye Res..

[15] Treharne A.J., Grossel M.C., Lotery A.J., Thomson H.A. (2011). The chemistry of retinal transplantation: The influence of polymer scaffold properties on retinal cell adhesion and control. Br. J. Ophthalmol..

[16] Dong Y., Yong T., Liao S., Chan C.K., Stevens M.M., Ramakrishna S. (2010). Distinctive degradation behaviors of electrospun polyglycolide, poly (DL-lactide-co-glycolide), and poly(L-lactide-co-epsilon-caprolactone) nanofibers cultured with/without porcine smooth muscle cells. Tissue Eng. Part A.

[17] Da Silva G.R., Lima T.H., Oréfice R.L., Fernandes-Cunha G.M., Silva-Cunha A., Zhao M., Behar-Cohen F. (2015). In vitro and in vivo ocular biocompatibility of electrospun poly(ɛ-caprolactone) nanofibers. Eur. J. Pharm. Sci..

[18] Shahmoradi S., Yazdian F., Tabandeh F., Soheili Z.S., Hatamian Zarami A.S., Navaei-Nigjeh M. (2017). Controlled surface morphology and hydrophilicity of polycaprolactone toward human retinal pigment epithelium cells. Mater. Sci. Eng. C Mater. Biol. Appl..

[19] Liu P., Fu K., Zeng X., Chen N., Wen X. (2019). Fabrication and Characterization of Composite Meshes Loaded with Antimicrobial Peptides. ACS Appl. Mater. Interfaces.

[20] Sun Y., Han F., Zhang P., Zhi Y., Yang J., Yao X., Wang H., Lin C., Wen X., Chen J. (2016). A synthetic bridging patch of modified co-electrospun dual nano-scaffolds for massive rotator cuff tear. J. Mater. Chem. B.

[21] Liu P., Chen N., Jiang J., Wen X. (2019). New surgical meshes with patterned nanofiber mats. RSC Adv..

[22] Huang D., Lin C., Wen X., Gu S., Zhao P. (2016). A Potential Nanofiber Membrane Device for Filling Surgical Residual Cavity to Prevent Glioma Recurrence and Improve Local Neural Tissue Reconstruction. PLoS ONE.

[23] Thompson J.R., Worthington K.S., Green B.J., Mullin N.K., Jiao C., Kaalberg E.E., Wiley L.A., Han I.C., Russell S.R., Sohn E.H. (2019). Two-photon polymerized poly(caprolactone) retinal cell delivery scaffolds and their systemic and retinal biocompatibility. Acta Biomater..

[24] Beachley V., Wen X. (2010). Polymer nanofibrous structures: Fabrication, biofunctionalization, and cell interactions. Prog. Polym. Sci..

[25] Mousa S.A., Lorelli W., Campochiaro P.A. (1999). Role of hypoxia and extracellular matrix-integrin binding in the modulation of angiogenic growth factors secretion by retinal pigmented epithelial cells. J. Cell. Biochem..

[26] Mas-Moruno C., Fraioli R., Rechenmacher F., Neubauer S., Kapp T., Kessler H. (2016). αvβ3- or α5β1-Integrin-Selective Peptidomimetics for Surface Coating. Angew. Chem. Int. Ed..

[27] Hynes R. (2002). Integrins: Bidirectional, Allosteric Signaling Machines. Cell.

[28] Springer T., Dustin M. (2011). Integrin Inside-Out Signalling and the Immunological Synapse. Curr. Opin. Cell Biol..

[29] Mackay L., Khadra A. (2020). The bioenergetics of integrin-based adhesion, from single molecule dynamics to stability of macromolecular complexes. Comput. Struct. Biotechnol. J..

[30] Aisenbrey S., Zhang M., Bacher D., Yee J., Brunken W., Hunter D. (2007). Retinal Pigment Epithelial Cells Synthesize Laminins, Including Laminin 5, and Adhere to Them through 3- and 6-Containing Integrins. Investig. Ophthalmol. Vis. Sci..

[31] Afshari F., Kwok J., Andrews M., Blits B., Martin K., Faissner A., Ffrench-Constant C., Fawcett J.W. (2010). Integrin activation or alpha9 expression allows retinal pigmented epithelial cell adhesion on Bruch’s membrane in wet age-related macular degeneration. Brain.

[32] Gullapalli V., Sugino I., Zarbin M. (2008). Culture-induced increase in alpha-integrin subunit expression in retinal pigment epithelium is important for improved resurfacing of aged human Bruch’s membrane. Exp. Eye Res..

[33] Zarbin M. (2003). Analysis of retinal pigment epithelium integrin expression and adhesion to aged submacular human Bruch’s membrane. Trans. Am. Ophthalmol. Soc..

[34] Oyama E., Takahashi H., Ishii K. (2017). Effect of amino acids near the RGD sequence on binding activities between alphaIIbbeta3 integrin and fibrinogen in the presence of RGD-containing synthetic peptides from elegantin and angustatin. Peptides.

[35] Jiang C., Zeng X., Xue B., Campbell D., Wang Y., Sun H., Xu Y., Wen X. (2019). Screening of pure synthetic coating substrates for induced pluripotent stem cells and iPSC-derived neuroepithelial progenitors with short peptide based integrin array. Exp. Cell Res..

[36] Freitas V., Vilas Boas V., Pimenta D., Loureiro V., Juliano M., Carvalho M., Pinheiro J.J.V., Camargo A.C.M., Moriscot A.S., Hoffman M.P. (2007). SIKVAV, a Laminin α1-Derived Peptide, Interacts with Integrins and Increases Protease Activity of a Human Salivary Gland Adenoid Cystic Carcinoma Cell Line through the ERK 1/2 Signaling Pathway. Am. J. Pathol..

[37] Li X., Liu X., Josey B., Chou C.J., Tan Y., Zhang N., Wen X. (2014). Short Laminin Peptide for Improved Neural Stem Cell Growth. Stem Cells Transl. Med..

[38] Seiler M., Aramant R. (2012). Cell replacement and visual restoration by retinal sheet transplants. Prog. Retin. Eye Res..

[39] Lewallen M., Xie T. (2013). Cell-based therapies for retinal degenerative diseases: A thousand strategies. J. Glaucoma.

[40] Cruz L., Chen F., Ahmado A., Greenwood J., Coffey P. (2007). RPE transplantation and its role in retinal disease. Prog. Retin. Eye Res..

[41] Tezel T.H., Del Priore L.V. (1997). Reattachment to a substrate prevents apoptosis of human retinal pigment epithelium. Graefes Arch. Clin. Exp. Ophthalmol..

[42] Tezel T.H., Del Priore L.V. (1999). Repopulation of different layers of host human Bruch’s membrane by retinal pigment epithelial cell grafts. Investig. Ophthalmol. Vis. Sci..

[43] Gullapalli V., Sugino I., Patten Y., Shah S., Zarbin M. (2005). Impaired RPE survival on aged submacular human Bruch’s membrane. Exp. Eye Res..

[44] Raudszus B., Mulac D., Langer K. (2018). A new preparation strategy for surface modified PLA nanoparticles to enhance uptake by endothelial cells. Int. J. Pharm..

[45] Liao G.M., Yang C.C., Hu C.C., Teng L.W., Hsieh C.H., Lue S.J. (2018). Optimal loading of quaternized chitosan nanofillers in functionalized polyvinyl alcohol polymer membrane for effective hydroxide ion conduction and suppressed alcohol transport. Polymer.

[46] Nishiyabu R., Iizuka S., Minegishi S., Kitagishi H., Kubo Y. (2017). Surface modification of polyvinyl alcohol sponge with functionalized boronic acid to develop porous materials for multicolor emission, chemical sensing and 3D cell culture. Chem. Commun..

[47] Shiohara A., Prieto-Simon B., Voelcker N.H. (2021). Porous polymeric membranes: Fabrication techniques and biomedical applications. J. Mater. Chem. B.

[48] Kim J., Park J.Y., Kong J.S., Lee H., Won J.Y., Cho D.W. (2021). Development of 3D Printed Bruch’s Membrane-Mimetic Substance for the Maturation of Retinal Pigment Epithelial Cells. Int. J. Mol. Sci..

[49] Karampelas M., Sim D., Keane P., Papastefanou V., Sadda S., Tufail A., Dowler J. (2013). Evaluation of retinal pigment epithelium-Bruch’s membrane complex thickness in dry age-related macular degeneration using optical coherence tomography. Br. J. Ophthalmol..

[50] Andriu A., Crockett J., Dall’Angelo S., Piras M., Zanda M., Fleming I.N. (2018). Binding of alphavbeta3 integrin-specific radiotracers is modulated by both integrin expression level and activation status. Mol. Imaging Biol..

[51] Fang I.M., Yang C.H., Yang C.M., Chen M.S. (2008). Overexpression of integrin alpha (6) and beta (4) enhances adhesion and proliferation of human retinal pigment epithelial cells on layers of porcine Bruch’s membrane. Exp. Eye Res..

[52] Ivins J.K., Yurchenco P.D., Lander A.D. (2000). Regulation of neurite outgrowth by integrin activation. J. Neurosci..

[53] Arulmoli J., Wright H.J., Phan D.T.T., Sheth U., Que R.A., Botten G.A., Keating M., Botvinick E.L., Pathak M.M., Zarembinski T.I. (2016). Combination scaffolds of salmon fibrin, hyaluronic acid, and laminin for human neural stem cell and vascular tissue engineering. Acta Biomater..

[54] Mitchell A., Drinnan C.T., Jensen T., Finck C. (2017). Production of high purity alveolar-like cells from iPSCs through depletion of uncommitted cells after AFE induction. Differentiation.

[55] Cheng B., Yan Y., Qi J., Deng L., Shao Z.W., Zhang K.Q., Li B., Sun Z., Li X. (2018). Cooperative Assembly of a Peptide Gelator and Silk Fibroin Afford an Injectable Hydrogel for Tissue Engineering. ACS Appl. Mater. Interfaces.

[56] Phelan M.A., Kruczek K., Wilson J.H., Brooks M.J., Drinnan C.T., Regent F., Gerstenhaber J.A., Swaroop A., Lelkes P.I., Li T. (2020). Soy Protein Nanofiber Scaffolds for Uniform Maturation of Human Induced Pluripotent Stem Cell-Derived Retinal Pigment Epithelium. Tissue Eng. Part C Methods.

